# Clinical and Epidemiological Characteristics of COVID-19 Patients with SARS-CoV-2 Re-Detected on PCR Test after Discharge from Isolation

**DOI:** 10.3390/clinpract11040110

**Published:** 2021-12-18

**Authors:** Abdullah J. Alsahafi, Manal M. Al Daajani, Ahmed A. Osman, Abdulhamed L. Moawwad, Abdullah M. Algarni, Ibrahim M. Asiri, Wadea O. Nofal, Rayan M. Alselami

**Affiliations:** 1 Public Health Department, Jeddah Health Affairs Directorate, Ministry of Health, Jeddah 21577, Saudi Arabia; ajal-sahafi@moh.gov.sa (A.J.A.); manal-m-daajani@hotmail.com (M.M.A.D.); Dr.moawwad@gmail.com (A.L.M.); amalgarni@moh.gov.sa (A.M.A.); imasseri@moh.com (I.M.A.); wnofal@moh.gov.sa (W.O.N.); rmalselami@moh.gov.sa (R.M.A.); 2 Department of Health Education and Health Promotion, Faculty of Public Health and Health Informatics, Umm Al-Qura University, Makkah 24243, Saudi Arabia

**Keywords:** SARS-CoV-2, COVID-19, Kingdom of Saudi Arabia, real-time polymerase chain reaction, repeat positive

## Abstract

There have been multiple reports of patients with coronavirus disease (COVID-19) testing positive for severe acute respiratory syndrome coronavirus 2 (SARS-CoV-2) after discharge; however, information on the characteristics of such cases is limited. In this case report, we aimed to identify clinical and epidemiological characteristics of patients who had a repeat positive polymerase chain reaction (PCR) test for SARS-CoV-2. We analyzed data of 22 COVID-19 patients who tested positive for SARS-CoV-2 on polymerase chain reaction (PCR) testing after two consecutive negative PCR results following discharge from hospitals. The interval between the two positive tests in the episodes of COVID-19 ranged from 4 to 117 days. More than one-third of the cases were healthcare workers (HCWs) and one-third of them had comorbidities. The main symptoms were cough and fever, and we noticed that males experienced more symptoms and signs of COVID-19 than females. Individuals with repeat SARS-CoV-2 positivity tend to experience milder illness during the second episode than the first episode. To confirm the reinfection of SARS-CoV-2, the results of other tests, such as viral culture and immunological assays of immunoglobulin G (IgG) and immunoglobulin M (IgM), need to be considered. Recovered COVID-19 patients should continue social distancing, using face masks, and practicing hand hygiene, especially HCWs who are more likely to be exposed to SARS-CoV-2.

## 1. Introduction

On 31 December 2019, the World Health Organization (WHO, Geneva, Switzerland) Country Office in China reported a case of pneumonia of unknown cause in Wuhan, China. On 30 January 2020, the WHO declared the outbreak a Public Health Emergency of International Concern. The new disease was subsequently named coronavirus disease (COVID-19), and the causative virus was identified as severe acute respiratory syndrome coronavirus 2 (SARS-CoV-2) [1]. COVID-19 spread rapidly worldwide and became a pandemic. As of 10 December 2021, over 267 million confirmed cases and over 5 million deaths in 216 countries have been reported to the WHO, and the number of cases is still rapidly increasing [2]. 

In the Kingdom of Saudi Arabia (KSA), patients with COVID-19 who require admission to hospitals are discharged after they have been asymptomatic for more than three days and have at least two consecutive negative SARS-CoV-2 results on real-time polymerase chain reaction (RT-PCR) tests [3]. Several scientists have raised concern regarding the possibility of SARS-CoV-2 reinfection [4,5,6]. Additionally, the clinical course and laboratory results of patients who have repeat positive tests for SARS-CoV-2 have not been well-documented. The Ministry of Health has imposed curfews and published guidelines to control the pandemic [3]. There have been reports from multiple countries of confirmed COVID-19 cases becoming SARS-CoV-2-positive after discharge. Our study explored the clinical and epidemiological characteristics of patients who had a repeat positive test for SARS-CoV-2 after recovery. In the KSA, no studies have determined whether repeat positive results are due to reinfection. This case report could be the first study of multiple cases with SARS-CoV-2 reinfection in the KSA. Our hypothesis was that repeat positive tests indicated reinfection. There were several concerns in the KSA regarding the possibility of SARS-CoV-2 reinfection. Therefore, we aimed to explore the clinical and epidemiological characteristics of such patients.

## 2. Materials and Methods

In this case report study, we included all admitted patients who tested positive for SARS-CoV-2 after recovering from COVID-19 [3]. We excluded patients who had positive tests within 2 days after negative test results, as well as those with unconfirmed or invalid laboratory tests. Overall, 22 patients met our inclusion criteria and were included in our study. The national guidelines recommended the discharging of patients after three days without symptoms and two negative respiratory samples separated by at least 24 h after the last positive PCR test. The WHO guidelines on discharging patients from isolation recommend the discharge of symptomatic patients after 10 days of symptom onset with more than 3 days free of symptoms without a need to retest. The guidelines recommend discharging asymptomatic patients 10 days after testing positive for SARS-CoV-2 [7]. We included all repeat positive patients who met the national guidelines criteria for discharging COVID-19 patients from the start of the outbreak in January 2020 until August 2020 in Jeddah, KSA. We traced patients retrospectively to identify their clinical and epidemiological characteristics. Laboratory confirmation of SARS-CoV-2 infection was obtained by RT-PCR assays of nasopharyngeal swabs, in line with the recommendation of the national guidelines. We obtained data from active and passive surveillance records of hospitals and the Public Health Department in Jeddah Health Affairs. We also used the Health Electronic Surveillance Network and hospital records to have direct telephonic communication with the included patients. The data collected included patient characteristics such as age, sex, occupation, nationality, presence of comorbidities (diabetes, hypertension, and asthma), signs and symptoms of COVID-19, duration and severity of COVID-19, and interval between the first and second episodes of COVID-19.

In this study, we used BGI’s Real-Time Fluorescent RT-PCR Kit for detecting SARS-CoV-2. It is one of the kits used characterized by a highly sensitive SARS-CoV-2 detection rate. Samples of the patients were collected from the throat (oropharyngeal) swabs and nasopharyngeal swabs by using the MGI automated sample preparation system. The laboratories run the test on the Roche LightCycler 480 Instrument as well as Applied Biosystems 7500 Fast, 7500, and QuantStudio 5 Real-Time PCR Systems. BGI’s Real-Time Fluorescent RT-PCR Kit deals with Taqman Reverse Transcription PCR and it targets the ORF1ab gene as the domain target. It is highly sensitive, detecting as low as 100 viral copies/mL for BALF samples, and highly specific, with no cross-reactivity with 54 human respiratory pathogens.

### Statistical Analysis

Data analysis was performed using SPSS version 21 (IBM Corp, Armonk, NY, USA). Distributions were summarized using descriptive statistics and are presented as frequencies. Categorical variables are summarized as frequencies and proportions. The Shapiro–Wilk test was used to test for normality of the distribution of continuous variables (age, illness duration, and interval time between the first and second episodes). The values of the Shapiro–Wilk test were less than 0.05, indicating that the data were not normally distributed. Hence, we measured median values and corresponding interquartile ranges (IQRs) to summarize continuous variables. The comparisons between study groups were performed using the Chi-squared test and Fisher’s exact test, as appropriate. *p*-values < 0.05 were considered statistically significant.

## 3. Results

**Figure 1:**
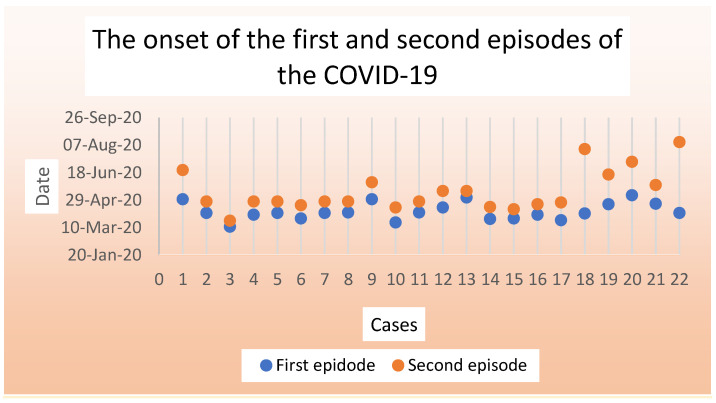
The onset of the first and second episodes of COVID-19.

A total of 22 patients who were reinfected with SARS-CoV-2 were included in this study. The median age was 33.5 years (range: 23–57 years). The median durations from illness onset to cure in the first episodes were 12.5 days (range: 8–38 days), whereas the median interval between first and second episodes was 11.5 days (range: 4–117 days) (Table 1 and Table 2). The onset of the first and second episodes of the COVID-19 is illustrated in (Figure 1 and Table S1). Most of the patients (64% (14/22)) were male, 50% (11/22) were Saudi nationals, 36% (8/22) were healthcare workers (HCWs), such as doctors, nurses, and physiotherapists, and 32% (7/22) had comorbidities such as diabetes, hypertension, and asthma (Table 1 and Table 2).

Cough and fever were the most common symptoms experienced by the patients. From the present findings, we noticed that males experienced more symptoms and signs of COVID-19 than females, and they had more chest X-ray abnormalities, as well as they needed antibiotic therapy and oxygen support more than females. Although, the differences between males and females were not statistically significant and this may be attributed to the small sample size (Table 3).

Fewer patients experienced fever during the second episode compared to the first episode. During the first episode, 36% (8/22) of patients required antiviral therapy, 41% (9/22) required antibiotic therapy, and 23% (5/22) required supplemental oxygen (Table 4). Generally, the second episode of COVID-19 was asymptomatic or milder than the first episode. All patients (100%) were symptomatic during the first episode compared to 9 patients (41%) during the second episode. In addition, only 3 patients (14%) needed hospital admission during the second episode compared to 13 patients (59%) during the first episode. Moreover, most of the patients with repeat positive results had no symptoms or mild symptoms during the second episode; thus, no therapeutic intervention was required. In this study, we noticed that males had a relatively higher severity of the COVID-19 than females. This observation is manifested by the number of male patients admitted in the hospital (9:4, 2:1) in the first and second episodes, respectively, as well as the number of males who needed oxygen support (4:1) (Table 4).

## 4. Discussion

In this study, we reported about 22 patients who had repeat positive PCR tests for SARS-CoV-2 after 2 consecutive negative test results over a period of 8 months in Jeddah Health Affairs hospitals. The interval between the first negative result at the end of the first episode and the first positive result at the beginning of the second ranged from 4 to 117 days. More than one-third of the patients who had repeat positive tests were HCWs, and more than one-third of the patients had comorbidities. The second episode was either asymptomatic or milder than the first episode. The main symptoms were cough and fever.

Viral reinfection is not uncommon, and it occurs with several respiratory viral infections. Moreover, reinfection with respiratory viruses may be explained by an insufficient immune response or infection with another genotype or species of virus as well as the diversity of viral serotyping [8]. There have been several reports from multiple countries of patients having repeat positive tests for SARS-CoV-2 after recovering from COVID-19 [8,9,10,11,12,13,14,15]. A case report from South Korea identified 163 patients who had repeat positive test results for SARS-CoV-2 [15]. A case report from China found a confirmed case of COVID-19 turned positive for SARS-CoV-2 RNA in convalescence on an oropharyngeal swab test [9]. Another study conducted by Lan et al. in Wuhan, China, reported about four medical professionals who tested positive for SARS-CoV-2 on RT-PCR tests 5–13 days after recovery, and suggested that some recovered patients may remain convalescent carriers [10]. Yujian et al. conducted a study among seven patients from China who had been discharged from a hospital but tested positive on RT-PCR tests 7–11 days after discharge [11].

In this study, the age of the participants ranged from 23 to 57 years, as reported by various studies [12,13,14]. We noticed that HCWs had a relatively high rate of repeat positive tests. This may be due to the prolonged exposure to the virus within their work environments [5,16]. In addition, approximately one-third of the patients who had repeat positive tests had other comorbidities, such as diabetes, hypertension, and asthma. One study found a high prevalence of comorbidities among patients with repeat positive tests [17].

The maximum duration of positivity during the first episode was 38 days, which reflects the burden that COVID-19 places on the healthcare system, as well as a possibility of an increased infection rate among contacts of positive patients. The maximum interval between the first and second episodes was 117 days, suggesting the possibility of reinfection. Cough and fever were the most common symptoms experienced by patients during the first episode. Additionally, the second episode of positivity was asymptomatic or milder than the first episode, and fewer patients required hospital admission during the second episode.

Several patients were retested despite the absence of symptoms. Many patients requested retesting for reassurance and to avoid a false-negative result. RT-PCR tests may produce false-negative results owing to low sensitivity, inadequate diagnostic timing, or low viral load [18]. In addition, some patients may have been inaccurately diagnosed with COVID-19 based on real-time RT-PCR tests having false-positive results [19,20,21]. There are concerns about the accuracy of PCR tests in detecting the active strain or portion of the virus at the time of taking a sample for testing. There have been several cases of reinfection with SARS-CoV-2 reported in other countries [8,12,13].

The interval between the first and second episodes of COVID-19 extended up to 117 days. One study could not clarify whether the repeat positive PCR test result represented a new infection or long-term viral shedding [4]. In our study, 18% of the patients had symptoms and a relatively long interval (1 month or more) between the first and second episodes; therefore, there is a possibility that they were reinfected with SARS-CoV-2. Some researchers consider an interval of 1 month between the first and second episodes as a new SARS-CoV-2 infection [6]. To confirm the occurrence of reinfection among patients, the RT-PCR test should be accompanied by other confirmatory laboratory tests, such as immunological assays of immunoglobulin G (IgG) and immunoglobulin M (IgM). One of the study limitations is the description of the cases of repeat positivity reported from hospitals, which may not reflect the actual number of repeat cases in the KSA. Hence, further studies are needed to determine the incidence of repeat positive PCR results and link those results with other confirmatory laboratory tests, such as viral culture and immunological assays of IgG and IgM, to distinguish between prolonged viral shedding and SARS-CoV-2 reinfection.

## 5. Conclusions

This study illustrates that many patients who have recovered from COVID-19 have repeat positive PCR tests for SARS-CoV-2 after having negative results with or without symptoms. The severity of the second episode is generally milder than the first episode. Many of the repeat positive cases occurred in HCWs and patients who had other comorbidities. It indicates that the policy of discharging patients with COVID-19 should be reconsidered, and RT-PCR test results should be combined with other tests such as viral culture and immunological assays of IgG and IgM, if possible. We recommend the continuation of social distancing, use of face masks, proper hand hygiene, and other preventive measures even in recovered patients, especially in HCWs who are at higher risk of reinfection as they are more likely to be exposed to SARS-CoV-2. Further studies should be conducted to explore the association between the repeat positive tests and the immunological status of patients in terms of the presence of IgG and IgM.

## Figures and Tables

**Table 1:** Demographic characteristics of the participants (*n* = 22).

Character	Minimum	Maximum	Median (IQR)	*p*-Value
Age	23	57	33.5 (17)	0.04 *
Duration of illness to cure (first episode)	8	38	12.5 (8)	0.000 *
Interval between first and second episode	4	117	11.5 (18)	0.000 *

* Shapiro–Wilk test.

**Table 2:** The demographic features and characteristics of the participants (*n* = 22).

	Male (*n* = 14) *n* (%)	Female (*n* = 8) *n* (%)	All (*n* = 22) *n* (%)	*p*-Value
Sex				
Male	14 (100)		14 (64)	
Female		8 (100)	8 (36)	
Nationality				
Saudi	6 (43)	5 (62.5)	11 (50)	0.659 **
Non-Saudi	8 (57)	3 (37.5)	11 (50)	
Occupation				
HCW	2 (14)	6 (75)	8 (36)	0.002 ***
Non-HCW	12 (86)	2 (25)	14 (64)	
Comorbidities ^a^				
Present	4 (29)	3 (37.5)	7 (32)	1.000 **
Absent	10 (71)	5 (62.5)	15 (68)	

^a^ Comorbidities included diabetes mellitus, hypertension, and asthma. HCW, healthcare worker. ** Fisher’s Exact Test. *** Chi-square Test.

**Table 3:** Symptoms, presence of chest X-ray abnormalities, and treatment of the participants (*n* = 22).

	Male (*n* = 14) *n* (%)	Female (*n* = 8) *n* (%)	All (*n* = 22) *n* (%)	*p*-Value
First episode				
Cough	12 (86)	8 (100)	20 (91)	0.515 *
Fever	12 (86)	5 (62.5)	17 (77)	0.309 *
Shortness of breath	3 (21)	2 (25)	5 (23)	1.000 *
Headache	2 (14)	1 (12.5)	3 (14)	1.000 *
Myalgia or malaise	4 (29)	1 (12.5)	5 (23)	0.613 *
Gastrointestinal symptoms	1 (7)	2 (25)	3 (14)	0.527 *
Sore throat and/or runny noise	7 (50)	5 (62.5)	12 (54.5)	0.675 *
Chest X-ray abnormalities	3 (21)	2 (25)	5 (23)	1.000 *
Use of antiviral therapy (oseltamivir)	4 (29)	4 (50)	8 (36)	0.386 *
Use of antibiotic therapy	6 (43)	3 (37.5)	9 (41)	1.000 *
Oxygen support	4 (29)	1 (12.5)	5 (23)	0.613 *
Second episode				
Fever	3 (21)	4 (50)	7 (32)	0.343 *

* Fisher’s Exact Test.

**Table 4:** Illness severity during the first and second episodes (*n* = 22).

Episode	Illness Severity	Male (*n* = 14) *n* (%)	Female (*n* = 8) *n* (%)	All (*n* = 22) *n* (%)	*p*-Value
First episode	Patients with symptoms	14 (100)	8 (100)	22 (100)	-- *
	Mild	5 (36)	4 (50)	9 (41)	0.646 **
	Moderate	8 (57)	4 (50)	12 (54.5)	
	Severe	1 (7)	0 (0)	1 (4.5)	
	Hospital admission	9 (64)	4 (50)	13 (59)	0.662 ***
Second episode	Patients with symptoms	5 (36)	4 (50)	9 (41)	0.662 ***
	Mild	3 (21)	3 (37.5)	6 (27)	0.715 **
	Moderate	2 (14)	1 (12.5)	3 (14)	
	Severe	0 (0.0)	0 (0.0)	0 (0.0)	
	Hospital admission	2 (14.3)	1 (12.5)	3 (14)	1.000 ***

* *p*-value not computed (all patients developed symptoms in the first episode). ** Chi-square Test. *** Fisher’s Exact Test.

## Data Availability

Not applicable.

## Supplementary Materials

The following are available online at https://www.mdpi.com/article/10.3390/clinpract11040110/s1, Table S1: SARS-CoV-2 PCR test results by participant and date (*n* = 22).
