# Supplementary material for: Clinical and Epidemiological Characteristics of COVID-19 Patients with SARS-CoV-2 Re-Detected on PCR Test after Discharge from Isolation
Source: Clin Pract. 2021 Dec 18;11(4):954–60. doi: 10.3390/clinpract11040110 (PMC8699873; doi:10.3390/clinpract11040110)
Supplement: Supplementary file 1 [file clinpract-11-00110-s001.zip › clinpract-1456124-supplementary.pdf]

Table S1. SARS-CoV-2 PCR test results by participant and date (N = 22).

Case ID	Symptoms	Result	Date	Interval (days)[a]	Case ID	Symptoms	Result	Date	Interval (days)[a]
1	Yes	+1[b]	1-May-20	44	9	Yes	1	1-May-20	12
1	No	−	10-May-20		9	No	−	9-May-20	
1	No	−	14-May-20		9	No	−	12-May-20	
1	Yes	+2[d]	23-Jun-20		9	Yes	2	21-May-20	
2	Yes	1	6-Apr-20	9	9	Yes	2	1-Jun-20	
2	Yes	1	13-Apr-20		10	Yes	1	20-Mar-20	23
2	No	−	18-Apr-20		10	No	−	24-Mar-20	
2	No	−	20-Apr-20		10	No	−	29-Mar-20	
2	Yes	2	27-Apr-20		10	Yes	2	16-Apr-20	
3	Yes	1	12-Mar-20	4	11	Yes	1	7-Apr-20	9
3	No	1	17-Mar-20		11	Yes	1	13-Apr-20	
3	No	−	19-Mar-20		11	No	−	18-Apr-20	
3	No	−	21-Mar-20		11	No	−	20-Apr-20	
3	No	2	23-Mar-20		11	Yes	2	27-Apr-20	
4	Yes	1	3-Apr-20	9	12	Yes	1	16-Apr-20	8
4	Yes	1	6-Apr-20		12	No	1	26-Apr-20	
4	Yes	1	8-Apr-20		12	No	−	30-Apr-20	
4	No	1	13-Apr-20		12	No	−	4-May-20	
4	No	−	18-Apr-20		12	Yes	2	8-May-20	
4	No	−	20-Apr-20		12	Yes	−	12-May-20	
4	No	2	27-Apr-20		12	No	2	16-May-20	
5	Yes	1	6-Apr-20	14	13	Yes	1	4-May-20	4
5	Yes	−	13-Apr-20		13	No	−	12-May-20	
5	No	−	18-Apr-20		13	No	−	13-May-20	
5	No	−	20-Apr-20		13	No	2	16-May-20	
5	No	2	27-Apr-20		14	Yes	1	26-Mar-20	8
6	Yes	1	27-Mar-20	6	14	No	−	2-Apr-20	
6	Yes	1	28-Mar-20		14	No	1	5-Apr-20	
6	Yes	1	8-Apr-20		14	No	−	9-Apr-20	
6	No	−	14-Apr-20		14	No	−	13-Apr-20	
6	No	−	17-Apr-20		14	No	2	17-Apr-20	
6	No	2	20-Apr-20		15	Yes	1	27-Mar-20	7
7	Yes	1	6-Apr-20	14	15	Yes	1	31-Mar-20	
7	Yes	−	13-Apr-20		15	No	−	6-Apr-20	
7	No	−	18-Apr-20		15	No	−	9-Apr-20	
7	No	−	20-Apr-20		15	No	2	13-Apr-20	
7	No	2	27-Apr-20		16	Yes	1	3-Apr-20	11
8	Yes	1	7-Apr-20	9	16	Yes	1	7-Apr-20	
8	No	1	13-Apr-20		16	No	−	11-Apr-20	
8	No	−	18-Apr-20		16	No	−	15-Apr-20	
8	No	−	20-Apr-20		16	No	2	22-Apr-20	
8	No	2	27-Apr-20		20	Yes	1	8-May-20	41
17	Yes	+1[b]	24-Mar-20	14	20	No	−	19-May-20	
17	Yes	1	1-Apr-20		20	No	−	21-May-20	
17	No	−	7-Apr-20		20	No	−	26-Jun-20	
17	No	−	12-Apr-20		20	Yes	2	29-Jun-20	

17	No	+2[d]	21-Apr-20	
17	No	2	25-Apr-20	
18	Yes	1	5-Apr-20	105
18	No	−	11-Apr-20	
18	No	−	15-Apr-20	
18	Yes	2	24-Jul-20	
18	No	2	31-Jul-20	
19	Yes	1	22-Apr-20	30
19	Yes	−	23-Apr-20	
19	No	−	7-May-20	
19	Yes	2	23-May-20	
19	No	2	8-Jun-20	
19	No	2	15-Jun-20	
20	No	2	8-Jul-20	
21	Yes	1	23-Apr-20	25
21	No	−	2-May-20	
21	No	−	4-May-20	
21	Yes	2	27-May-20	
22	Yes	1	6-Apr-20	117
22	Yes	1	13-Apr-20	
22	No	−	18-Apr-20	
22	No	−	20-Apr-20	
22	No	−	9-Jun-20	
22	Yes	2	13-Aug-20	

[a] Interval period in days between first negative results in the first episode and first repeat-positive results in the second episode with period more than 2 days. [b]: Positive results of the first episode; −: Negative laboratory results; [d]: Positive results after negative one with period more than 2 days.
